# Pseudomonas fluorescens from Lake Bogoria, Kenya: a promising biocontrol agent against Fusarium solani in Phaseolus vulgaris L.

**DOI:** 10.1099/mic.0.001593

**Published:** 2025-08-11

**Authors:** Tofick B. Wekesa, Justus M. Onguso, Damaris Barminga, Ndinda Kavesu

**Affiliations:** 1 Institute for Biotechnology Research, Jomo Kenyatta University of Agriculture and Technology, P.O BOX 62 000 – 00200, Nairobi, Kenya; 2 Production, Novo Science Bio Solutions Limited, P.O BOX 39464-00623, Nairobi, Kenya, Kenya

**Keywords:** *Antibiosis genes*, biocontrol, *Fusarium solani*, *Phaseolus vulgaris *L., *Pseudomonas fluorescens*, soda lake

## Abstract

Common beans (*Phaseolus vulgaris* L) are an important staple crop valued for their high protein content and dietary benefits. However, Fusarium wilt, caused by *Fusarium solani*, is responsible for up to an 84% yield loss in bean production. This study aimed to isolate and evaluate novel *Pseudomonas fluorescens* from Lake Bogoria as potential biocontrol agents against *F. solani*. Using serial dilution, 30 bacterial isolates were obtained; 10 showed varied mycelial inhibition rates (5.95–42.86%) through dual culture and confrontation assays. Molecular identification using 16S rDNA confirmed that two isolates were *Pseudomonas fluorescens* strains. Antibiosis gene screening revealed the presence of 2,4-diacetylphloroglucinol, pyrrolnitrin, pyoluteorin and hydrogen cyanide. Enzyme assays demonstrated chitinase (1.33–3,160 U ml^−1^) and chitosanase (12.67–29.00 mm) production, indicating antifungal capabilities. *In vivo* pot experiments with isolate TW17^+^ showed reduced wilt symptoms <20.0% and disease incidence (8.0–35.0%). These findings highlight the potential of soda lake-derived *Pseudomonas fluorescens* as an effective biocontrol agent against *F. solani*, with additional benefits for common bean growth and yield improvement.

## Data Availability

Data used to support the findings of this study are available from the corresponding author upon request.

## Introduction

In Kenya, agriculture is considered the most prominent and dominant sector, contributing to up to 21.8% of the Gross Domestic Product (GDP) [1]. Within the agricultural sector, vegetable farming plays a crucial role in ensuring food security and improving the livelihoods of small-scale farmers. Common beans, particularly a vital crop within this sector (*Phaseolus vulgaris* L.), are a herbaceous annual crop renowned for its edible dry seed and green pods [2]. In Kenya, common beans are a staple food, second to maize, and are cherished for their nutritional value, high protein content and contribution to dietary diversification [3]. The leaves of these plants are also occasionally used as vegetables and fodder.

Additionally, they provide nutrients such as protein, minerals, fibre, thiamine, folate and phytochemicals with analgesic and neuroprotective properties [1]. Furthermore, they are rich in iron, zinc and other microelements that are typically deficient in cereal grains and root crops, positioning them as an excellent candidate for biofortification. Their high dietary fibre content promotes digestive health, regulates blood sugar levels and aids in weight management by inducing a sense of fullness and preventing overeating [4].

Economically, common beans are crucial cash crops for many smallholder farmers in Kenya [5]. It is a source of income and food and fosters overall economic development in the rural areas. Kenya exports common beans to various international markets, bolstering foreign exchange earnings and supporting the country’s balance of trade. The demand for Kenyan beans is significant due to their quality and taste [6]. Despite that, bean production does not sustain the demand, and this discrepancy is supplemented by imports from Uganda, Ethiopia and Tanzania [1]. This has significantly contributed to the increased prevalence of pests and diseases [7], with pests such as cutworms, bean flies, red spider mites, aphids, pod borers, whiteflies and thrips, as well as diseases like black root rot, damping-off, bean rust, Fusarium wilt, bacterial blight and downy mildew, posing serious threats to bean cultivation [8].

Soil infertility and environmental stress have intensified the challenges in common bean production in Kenya, contributing to a 13.8% decline over the past 5 years [9]. One of the most damaging diseases is root and stem rot (50–70% incidence), primarily caused by Fusarium fungi, particularly *Fusarium solani* [10]. This pathogen is difficult to identify and manage due to its ability to persist in the soil as chlamydospores, survive on decaying organic matter and spread through contaminated debris and runoff [11]. The disease typically presents as irregular light brown lesions on the stem, tap root and hypocotyl, which enlarge and darken over time [12,13]. *F. solani* penetrates plant roots through wounds or natural openings, leading to severe infections that can cause up to 70% yield loss in common beans, highlighting the urgent need for effective alternative management strategies [14,15]. Various strategies have been employed to manage Fusarium root rot, though their effectiveness varies. Synthetic fungicides are commonly used for seed treatment, but they generally show limited efficacy in controlling the disease. Ashraf *et al.* [16] reported that fungicides like prochloraz, bromuconazole and benomyl were most effective against *Fusarium oxysporum*; however, their use raises concerns due to the development of resistance and the harmful effects on non-target organisms. In addition to chemical control, farmers have adopted cultural practices such as crop rotation, which leverages climate adaptability, genetic diversity and sanitation [17,18]. Other approaches include adjusting sowing density and timing, selecting appropriate soil types [19], using resistant varieties or species mixtures [20] and practising selective weeding [21].

Disease management using biocontrol agents represents a potential alternative to synthetic fungicides and traditional practices. Additionally, biocontrol is compatible with pesticide-free agriculture and the environment. Some of the most promising biocontrol agents reported are *Bacillus velezensis* for biocontrol of *F. solani* [22] and *Bacillus subtilis* CAS 32 for biocontrol of *F. solani* causing root rot in beans [23]. *The Pseudomonas* genus has been reported to manage Fusarium diseases [24].

Additionally, the study by Gade and Koche [25] reported that *Pseudomonas* spp. strains enhance the suppression of *Fusarium* wilt in radish. The antagonistic activity of *Pseudomonas* spp. against *F. solani* has been investigated to assess their efficacy as a biocontrol agent and to elucidate their antagonistic mechanisms [26]. Through *in vitro* and *in vivo* assays, the antagonistic capacity of *Pseudomonas* against various plant pathogens has been demonstrated through mycoparasitism, induction of plant resistance, inhibitory metabolites production, plant growth stimulation, competition for nutrients and space and production of lytic enzymes [26,29].

One promising area of research involves the extremophilic bacteria found in Kenya’s soda lakes, such as Bogoria, Magadi and Elementaita [30]. These bacteria thrive in extreme environmental conditions and have potential applications in biocontrol strategies against pathogens *like F. solani*, affecting common beans [31]. By harnessing these extremophiles, sustainable and environmentally friendly solutions to enhance bean production and safeguard the livelihoods of Kenyan farmers can be achieved. Therefore, this study aimed to isolate, *in vitro* and *in vivo*, and evaluate *Pseudomonas* sp. from Lake Bogoria as a biocontrol agent against *F. solani*.

## Methods

### Description of the sampling site and sample collection

**Fig. 1.**
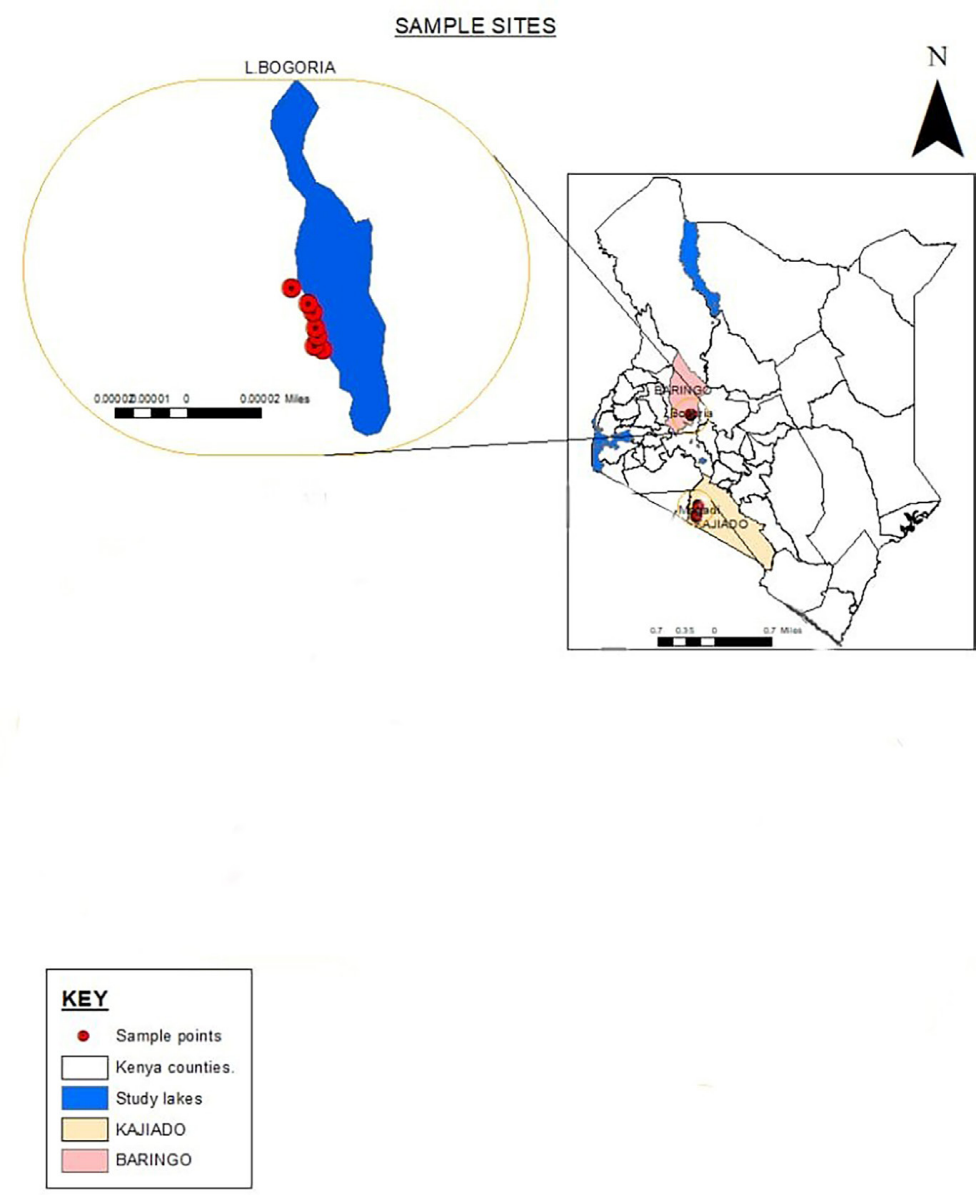
Kenyan map showing sampling points at Lake Bogoria.

Lake Bogoria is an alkaline saline lake in Baringo County, Kenya (longitude 36° 4′–36° 7′ E, latitude 0° 10′–0° 20′ N). The lake is a Ramsar site and has been protected as a national reserve since 2 November 1973. It is known for geysers and hot springs (>45.0 ℃) along the lake bank and is home to one of the world’s largest populations of lesser flamingos. Lake Bogoria is shallow, ~34 km long and 3.5 km wide. It also contains a large concentration of Na^+^, HCO_3_ and CO_3_^2-^ ions (pH >8.0). During the study, a purposive sampling method was used at six sampling points. Soil samples were collected in triplicate, with respective physicochemical characteristics recorded. The samples were separately packed in sterile tubes (Table 1, Fig. 1), stored in a cool box and transported to Jomo Kenyatta University of Agriculture and Technology for further analysis.

**Table 1.** Sampling points with their physicochemical characteristics

Sampling point	Latitude	Longitude	Elevation (m)	pH	Conductivity (mS cm^−1^)	Salt (ppt)	TDS (ppt)
BP01	N10°43.692	E058°25.621	1,009	9.6	6.0	2.9	28.6
BP02	N00°13.730	E036°05.572	1,009	9.2	25.6	12.1	17.0
BP03	N00°13.711	E036°05.575	1,010	9.6	10.0	10.1	13.4
BP04	N00°13.716	E036°05.577	1,008	8.8	6.1	2.9	3.9
BP05	N00°13.724	E036°05.584	1,007	8.7	5.9	3.1	4.1
BP06	N00°13.673	E036°05.603	1,007	8.2	26.1	13.0	17.2

TDS, Total Dissolved Solids.

### Isolation of *Pseudomonas* spp*.*

Isolation of *Pseudomonas* spp. from the soil was done as described by Atlas [32]. In summary, 1 g of soil samples was mixed with 9 ml of physiological sterile saline (0.85% NaCl) and then homogenized. The resulting suspensions were serially diluted in a ratio of 1:9 up to a dilution of 10^−4^. An aliquot of 30 µl was cultured on King’s B agar, HiMedia, India (20.0 g l^−1^ of proteose peptone, 1.5 g l^−1^ of dipotassium hydrogen phosphate, 1.5 g l^−1^ of magnesium sulphate heptahydrate and 10.0 g l^−1^ agar). Plates were incubated at 30℃ for 48 h, and colonies identical to *Pseudomonas* spp*.* that showed bluish-green fluorescence under UV light (254 nm) were sub-cultured and preserved for further analysis.

### Antibiosis assay of *Pseudomonas* spp*.* isolates against *F. solani*

The antibiosis assay of *Pseudomonas* spp. isolates against *F. solani* was carried out using a dual culture technique in Sabouraud dextrose agar (SDA), HiMedia (10.0 g l^−1^ peptones, 40.0 g l^−1^ dextrose monohydrate, 15.0 g l^−1^ agar) as described by Aydi *et al.* [33]. In summary, the bacteria isolate was streaked perpendicularly across the plate on a Potato Dextrose Agar (PDA) medium and a mycelium plug of an active 5-day growing *F. solani* ATCC 11712 (isolated from common beans and sourced from Dudutech| Bioline Agrosciences Africa, Naivasha, Kenya) was placed on the end side of the Petri dish. At the same time, the control was prepared by placing a mycelium plug on one side and sterile distilled water on the other side. The experiment had three replicates and was incubated at 30 ℃ for 14 days, and the length of the mycelium was measured using a ruler in centimetres (cm) with 1 mm accuracy.

### Molecular characterization of the selected bacterial isolates

Genomic DNA was extracted from the two isolates with the highest antagonistic activity against *F. solani* using a bacterial DNA isolation kit (Norgen Biotek Corp., Thorold, ON, Canada) according to the manufacturer’s instructions. The DNA was then quantified using Qubit (Qubit 4 Fluorometric quantification, Q33238), and its final concentration was adjusted to 100 ng of DNA per microlitre. PCR amplification of 16S rRNA genes was performed with universal primers fD1-5′AGA GTT TGA TCC TGG CTC AG 3′; rP2-5′. ACG GCT ACC TTG TTA CGA CTT 3′. The amplification was performed using a Peqlab Primus 96 thermocycler (PEQLAB, Erlangen, Germany) in a 40 µl reaction consisting of 20 µl of master mix, 18.2 µl of PCR water (10 p.p.m.), 0.4 µl of the fD1 primer (10 p.p.m.), 0.4 µl of the rP1 primer and 1.0 µl of template DNA (10 ng ml^−1^). The PCR conditions followed the cycling profiles as described by Wekesa *et al.* [22]. The PCR amplicons were then purified using the QIAquick PCR Amplification Kit (QIAGEN, Hilden, Germany) and sent to Macrogen (South Korea) for sequencing.

#### Phylogenetic analysis

The basic local alignment search tool (blast) from the National Center for Biotechnology Information (NCBI) site was used to compare the 16S rRNA of the bacterial isolates. The blast findings selected 16S rRNA gene sequences with the highest similarity index. The sequences were aligned using clustal w 2.0 and incorporated in mega 11 for pairwise and multiple sequence alignment [34]. Phylogenetic analysis was conducted using mega 11, where the Kimura two-parameter model was used to calculate the evolutionary distances and construct the phylogeny tree [34]. The evolutionary distance was calculated using the maximum composite likelihood techniques. A bootstrap analysis of 1,000 replicates denotes the evolutionary history of the already analysed taxa.

### Inoculum preparation and confrontation assay

#### Inoculum preparation

The bacterial strain TW17 and *F. solani* were used to determine the bacterial–fungal interaction. TW17 had the highest mycelium inhibition rate in the dual culture technique, which was cultured in Luria–Bertani (LB) broth, HiMedia (10.0 g l^−1^ tryptone, 5.0 g l^−1^ yeast extract and 10.0 g l^−1^ sodium chloride) at 30 ℃ in a shaker incubator at 120 r.p.m. for 24 h. 0.5 ml of the culture was subcultured in 50 ml of half-strength LB broth and incubated under previous conditions. The culture was preserved for confrontation assay as an inoculum.

The *F. solani* isolates were previously obtained from Dudutech Laboratory and identified through morphology and molecular analyses. The pathogenicity of this isolate was previously tested and confirmed in different studies [35]. The isolate was subcultured on SDA, HiMedia, at 28 °C for 7 days. The spores were harvested as described by Chakravarthi *et al.* [36]. The obtained spores were used as inoculum for mycophagus activity.

#### Confrontation assay/mycophagous activity

Mycophagus activity between TW17 and *Fusarium* was performed to determine the bacteria’s mycophagous capacity when in direct contact with the phytopathogen.

In the confrontation assay, three treatments were determined: T_1_, TW17 inoculum only; T_2_, *Fusarium* inoculum only used as control; and T_3_, TW17+F .S. as described by Báez-Astorga *et al.* [37]. Briefly, all treatments were incubated at 30 ℃ in a shaker incubator at 120 r.p.m. An aliquot of 200.0 µl was taken from each condition at 12, 24 and 48 h to determine the bacterial–fungal interaction under a light microscope as described by Báez-Astorga *et al.* [37]. In brief, 20.0 µl of the samples from 200.0 µl aliquots of different conditions and times were inoculated on the slide and fixed to heat and stained with 0.5 % safranin for 30 s. The slide was then washed with distilled water to remove excess safranin and air-dried. The bacterial–fungal interaction was then observed under a light microscope at ×40, and the number of hyphal segments of the TW17+F .S and F.S control treatments was determined.

### Screening for genes associated with antibiosis

To determine the antibiotic genes responsible for antagonism, TW17 bacteria were examined by PCR for the presence or absence of genes associated with antibiosis using specific gene primers (Table 2). Genes selected coded for 2,4-diacetyl phloroglucinol (2,4-DAPG), pyrrolnitrin, phenazine-1-carboxylic acid, pyoluteorin and hydrogen cyanide antibiotics. The primers were set in a PCR mixture with a total reaction volume of 25.0 µl consisting of 10.0 µl of master mix, 8.2 µl of PCR water (10 p.p.m.), 0.4 µl of the F primer (10 p.p.m.), 0.4 µl of the R primer and 1.0 µl of template DNA (10.0 ng ml^−1^). The conditions of the PCR amplification cycle were initial denaturation at 95 ℃ for 5 min followed by 35 cycles of denaturation at 95 ℃ for 1 min, annealing at 68 ℃/62 ℃ (Table 2) for 45 s, extension at 72 ℃ for 1 min and final extension at 72 ℃ for 10 min. The presence or absence of the PCR amplicon was checked by electrophoresis in 1% agarose gels stained with ethidium bromide for band visualization using a Digi Doc imaging system (UVP, Cambridge, UK).

**Table 2.** Target antibiosis genes that encode enzymes for the biosynthesis of various antibiotics in the TW17 strain

Target gene	Primer	Sequence	Annealing	Expected PCR product size	Reference
phlD(2,4-DAPG)	Phl2a Phl2b	GAGGACGTCGAAGACCACCA ACCGCAGCATCGTGTATGAG	62 °C	745	[57]
phzCD (phenazine-1-carboxylic acid)	PCA2a PCA3b	TTGCCAAGCCTCGCTCCAAC CCGCGTTGTTCCTCGTTCAT	68 °C	1,150	[57]
prnD (pyrrolnitrin)	PRND1 PRND2	GGGGCGGGCCGTGGTGATGGA YCCCGCSGCCTGYCTGGTCTG	68 °C	786	[58]
pltC (pyoluteorin)	PLTC1 PLTC2	AACAGATCGCCCCGGTACAGAACG AGGCCCGGACACTCAAGAAACTCG	68 °C	438	[58]
pltB (pyoluteorin)	PltBf PltBr	CGGAGCATGGACCCCCAGC GTGCCCGATATTGGTCTTGACC	68 °C	791	[59]
hcnBC (hydrogen cyanide)	Aca Acb	ACTGCCAGGGGCGGATGTGC ACGATGTGCTCGGCGTAC	62 °C	587	[60]

### Production of lytic enzyme activity

#### Chitinase activity

The ability of TW17 to produce the chitinase enzyme was determined using the fluorometric chitinase assay kit (Sigma, USA), according to the manufacturer. Chitinase enzyme was defined as the amount of enzyme required to produce 1 µmol of 4-methylumbelliferone from the substrate per minute. Furthermore, the activity of endochitinase and exochitinase was assayed using different substrates per chitinase assay kit. For exochitinase enzymes, *N*-acetyl-*β*-d-glucosaminide (GlcNAc) and N,N′-diacetyl-*β*-d-chitobioside (GlcNAc-2) were determined, and for endochitinase activity, trimer *β*-d-N,N′,N″-triacetylchitotriose (GlcNAc-3) was assayed according to the manufacturer’s instructions, and each enzyme activity was performed in three replicates.

#### Chitosanase activity

The TW17 strain was evaluated for its ability to produce chitosanase enzymes using chitosanase detection agar (CDA), as described by Fawzya and Chasanah [38]. In brief, TW17 bacteria (1.0×10^8^ c.f.u. ml^−1^) were inoculated on a white paper filter disc (6 mm) and air-dried with negative control (distilled water). Then, it was placed on CDA plates (three discs per plate) with three replicates and incubated at 28 °C for 48 h. Chitosanase activity was determined by the presence of a clear zone of inhibition formed around the disc inoculated by the bacteria strain.

### *In vivo* antagonistic potential of selected TW17 using pot assays

The *in vivo* assay of the TW17 bacteria strain was designed to evaluate its protective properties against root rot of *F. solani* and its concentration and persistence in the rhizosphere of common bean roots under the greenhouse conditions using the pot culture method as described by Wekesa *et al.* [39]. The inoculum of TW17 was prepared by culture technique in a flask with 100 ml of nutrient broth, HiMedia, at 28 °C for 36 h in a shaker incubator at 120 r.p.m. and adjusted to a final concentration of 1.0×10^9^ c.f.u. ml^−1^. The inoculum of *F. solani* was prepared by culture in a 250-ml flask in Potato Dextrose Broth (PDB), HiMedia, at 28 °C for 14 days and adjusted to a final concentration of 2.0×10^8^ spores ml^−1^. Common bean seeds (yellow beans) were surface sterilized with 70% ethanol for 30 s and 5% NaOCl for 5 min and rinsed three times with sterile distilled water (3 seeds/pot) in pots (28 cm by 40 cm) containing sterilized cocopeat (200 g/pot) medium and incubated for 35 days in a greenhouse without temperature control and under natural light conditions. The experiment was conducted in a completely randomized block design with three replications at each treatment. The treatments included T_1_-TW17+*F. solani* (*F. solani* inoculated 5 days before adding TW17), T_2_-*F. solani* only, T_3_-TW17 only and absolute control T_4_ (without pathogen and bacteria). Treatments with TW17 bacteria were applied under three conditions: the bacteria were at the root level after 7, 14 and 28 days. The treated and untreated TW17 were infected with *F. solani*, and the incidence of the disease was evaluated using a scale from 1 to 7, as described by Bock *et al.* [40]. For fungal infection, five common bean plants were infected by immersion of roots for 2 h in *F. solani* inoculum (2.0×10^8^ spores ml^−1^). The development of the disease was scored weekly as wilted or unwilted for up to 3 weeks, and the number of plants with symptoms of *F. solani* was recorded.

### Survival and population density of the TW17 strain in common bean roots

The survival and population density of the inoculated bacteria strain were determined as described by Moruzzi *et al.* [41]. Root samples were collected weekly from all treatments and placed in sterile distilled water (10 ml/10 mg of root tissue) and incubated in a rotary shaker for 2 h. An aliquot (200 µl) of the suspension was serially diluted to 10^10^ in ratio 1 : 9. An aliquot of 30 µl was cultured in King’s B medium (protease peptone #3 (Difco), 20 g l^−1^; K2HPO · 1.5 g l^−1^; MgSO4•7H2O · 1.5 g l^−1^; glycerol, 10 ml l^−1^) incubated at 25 ℃ for 48 h, and colonies were counted using UV light.

### Data analysis

The raw data from antagonistic and confrontation assays and the production of lytic enzymes were subjected to a normality test using the Shapiro–Wilk test to evaluate the normal distribution. The data were also subjected to a homogeneity of variance test to check the assumption of equal variances across the data. The data were then subjected to ANOVA to determine statistically significant differences. For means separation, the least significant difference (LSD) test was used as a post hoc analysis using SAS version 8.0 software. The percentage incidence and wilted plants were determined using a graphical presentation using GraphPad Prism 6.

## Results

### Isolation and antibiosis assay

**Fig. 2.**
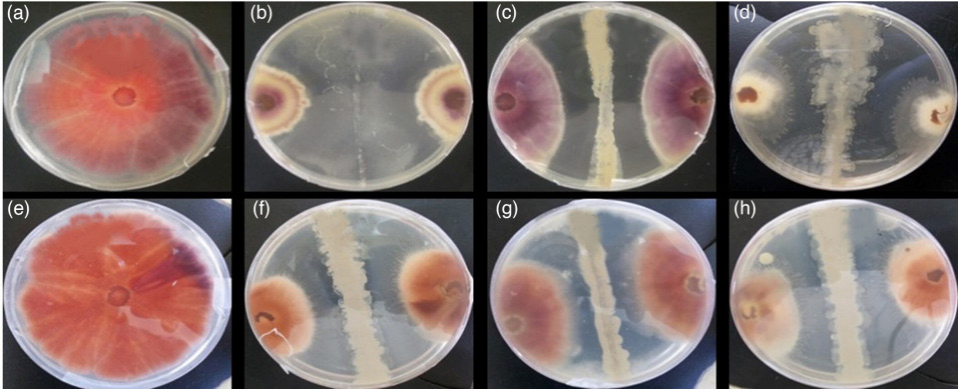
Antibiosis assay of positive Lake Bogoria bacterial isolates against *F. solani* after 14 days of incubation at 30 °C±2.0: (**a**) *F. solani* only, (**b**) TW21 and *F. solani*, (**c**) TW52 and *F. solani*, (**d**) TW19 and *F. solani*, (**e**) TW37 and *F. solani*, (**f**) TW50 and *F. solani* and (**g**) TW32 and *F. solani*.

The isolated bacterial colonies from Lake Bogoria soil samples were used in preliminary dual culture tests with *F. solani*. A total of 30 bacterial isolates were isolated from the different sampling sites of L. Bogoria. Among the 30 bacteria tested, 10 bacterial strains showed a varied mycelium inhibition rate (Table 3, Fig. 2). Of the ten bacteria, two bacteria (TW17, 42.86 %; TW19, 40.08 %) had the highest <40 % inhibition rate (Table 3, Fig. 2).

**Table 3.** Antagonistic activity of bacteria isolated against *F. solani*

Lake	Isolate code	Mycelium length (cm)	% Inhibition rate
**Bogoria**	Control	8.40±0.00^a^	0.00
	TW24	7.90±0.46^a^	5.95
	TW33	6.90±0.10^b^	17.86
	TW43	6.80±0.30^b^	19.05
	TW21	6.73±0.35^bc^	19.84
	TW32	6.63±0.29^bc^	21.03
	TW50	6.60±0.26^bcd^	21.43
	TW37	6.50±0.4^bcd^	22.62
	TW52	6.50±0.30^bcd^	22.62
	TW19	5.03±0.25^f^	40.08
	TW17	4.80±0.10^f^	42.86

The values indicate that the mean number of mycelium length from three biological replicates (*n*=3) with the same superscript letter(s) within the same column is not significantly different by Fisher’s test. According to Fisher’s LSD test (*P*<0.05), different superscript letters (a, b, c and d) indicate significantly different means within a column. Data are presented as mean±standard error.

### Molecular characterization of selected bacterial isolates

**Fig. 3.**
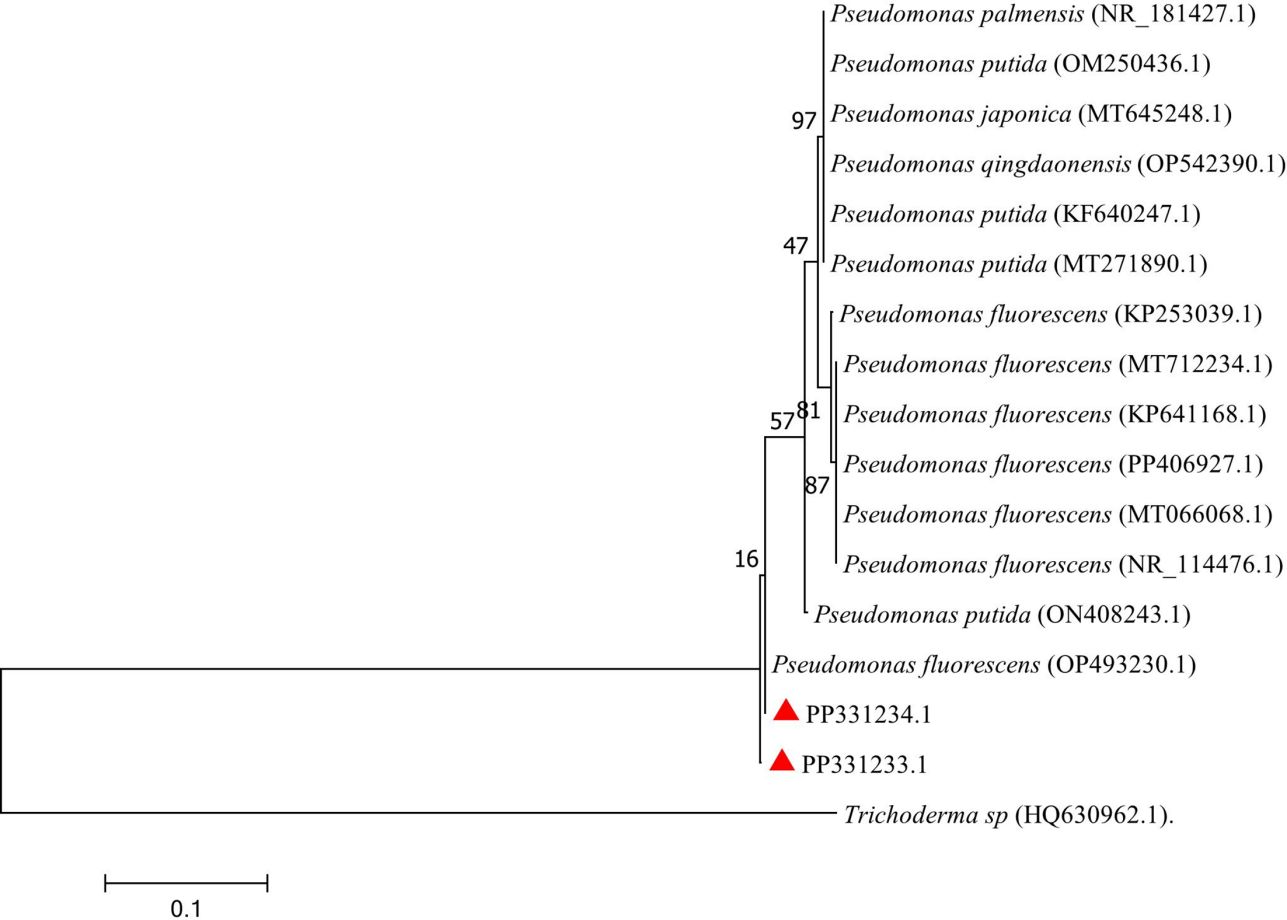
Molecular phylogenetic analysis by the maximum likelihood method. The evolutionary history was inferred by using the maximum likelihood method based on the Kimura two-parameter model. The tree is drawn to scale, with branch lengths measured in the number of substitutions per site. Evolutionary analyses were conducted in mega7.

Sequencing was performed on only two bacteria isolates with the highest >40.0 % inhibition rate from the dual culture technique. From the partial sequence, the blastn analysis showed that two isolates belong to the genus *Pseudomonas* (Table 4, Fig. 3). The two isolates were also submitted to NCBI GenBank and assigned accession numbers PP331233 and PP331234, respectively (Fig. 3). The bacteria strain TW17 was closely related to the *Pseudomonas fluorescens* with a percentage identity of 95.5% (Fig. 3). However, the bacterial strain TW19 was closely related to the strain of *Pseudomonas fluorescens* with a similarity index of 99.12% (Table 4).

**Table 4.** Molecular identification of selected antagonistic bacterial strains using 16S rRNA genes with the corresponding GenBank accession numbers

Bacterial strain	Accession no.	GenBank closest relative	Accession no.	Per. identity
**TW17**	PP331233	*Pseudomonas fluorescens*	NR_114476.1	95.5
**TW19**	PP331234	*Pseudomonas fluorescens*	OP493230.1	99.12

### Confrontation assay/mycophagous activity

**Fig. 4.**
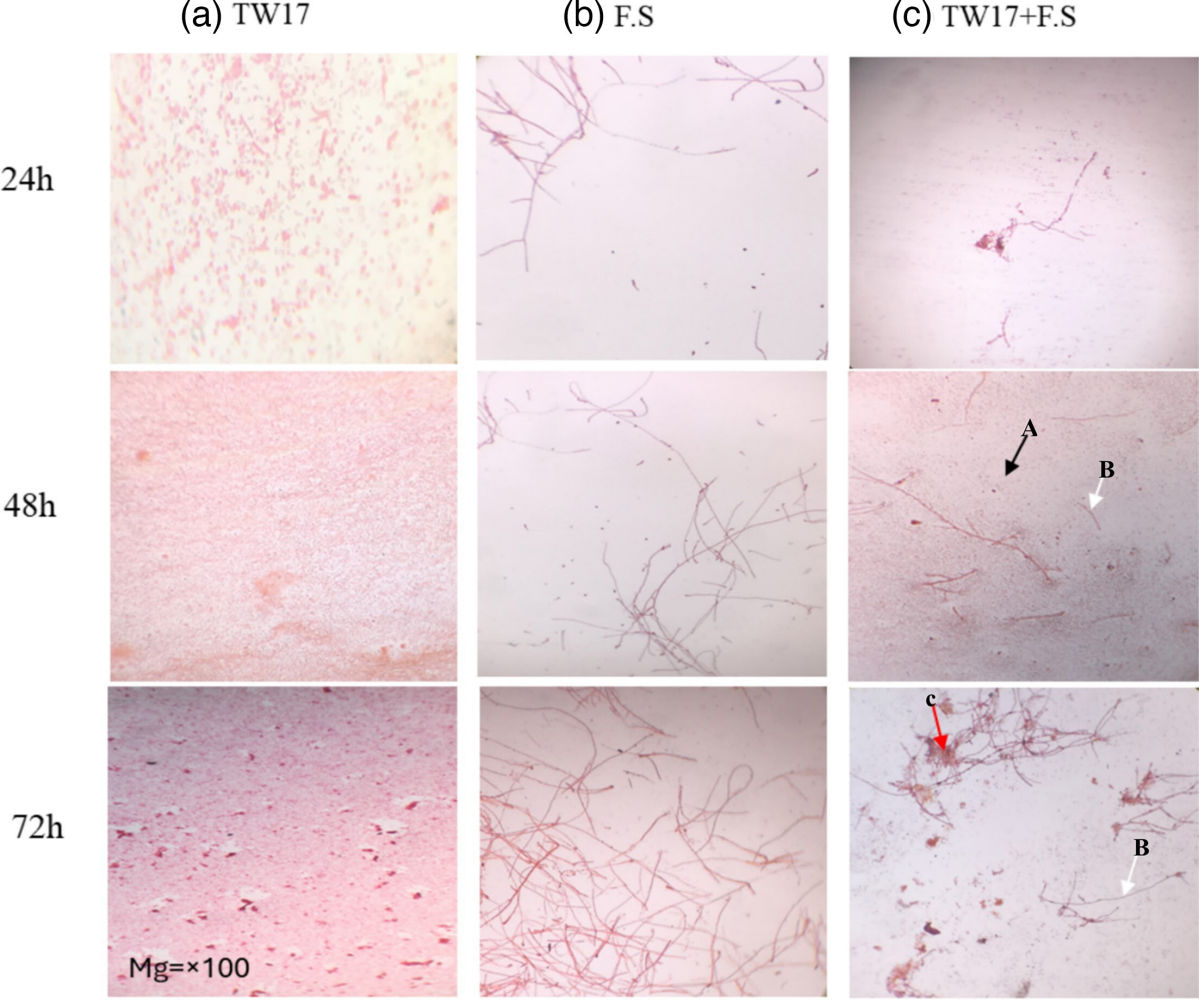
Microscopic visualization of samples from the confrontation test between strain TW17 and F.S in liquid culture. All samples were observed at 24, 48 and 72 h. Micrographs were taken under a light microscope at ×100. F.S, *Fusarium solani;* TW17, *Pseudomonas fluorescens*. (**a**) Bacterial cells, (**b**) mycelium (**c**) mycelium and bacteria.

The confrontation image from the light microscopes showed the inhibitory effect of strain TW17 on the growth of *F. solani* mycelium at 24 h, 48 h and 72 h in the TW17+F. solani treatment (Table 5). A lower branch count of fungal hyphae was observed after 24 h in treatment TW17+F .S. compared to the control. At 48 h, fungal hyphae in treatment TW17+F .S increased compared to 24-h and 72-h incubation. For the F.S. treatment, the fungal hyphae count increased throughout the incubation period. There was also an observation of cell wall thinning and swelling of the hyphae at 48 and 72 h in treatment with TW17+F .S. (Fig. 4).

**Table 5.** The number of branches of the mycelium of *F. solani* during the liquid confrontation assay between *F. solani* and *Pseudomonas fluorescens* TW17

Time (H)	No. of branches per *F. solani* hyphal segment	
	S	TW17+F .S
24	12.00±0.58^c^	4.00±0.58^e^
48	17.33±0.88^b^	6.67±0.33^d^
72	24.67±0.88^a^	3.33±0.33^e^
C.V	9.75	
LSD	1.97	
*P*>	0.0001	

The values indicate that the mean number of branches in five hyphals from three biological replicates (*n*=3) with the same superscript letter(s) within the same column is not significantly different by Fisher’s test. According to Fisher’s LSD test (*P*<0.05), different superscript letters (a, b, c and d) indicate significantly different means within a column. Data are presented as mean±standard error.

C.V, Co-effiency of Variance.

### Screening for antibiosis-associated genes

The set of primers used is involved in the biosynthesis of antibiotic metabolites against phytopathogens. Of the six genes investigated, five were detected during PCR amplification (Table 6). These include phlD for the synthesis of 2,4-diacetyl phloroglucinol, prnD for pyrrolnitrin, pltC and pltB for pyoluteorin and hcnBC to produce cyanide. Only phzCD genes for the production of phenazine-1-carboxylic acid were not detected. All PCR products were of the expected size (Table 2).

**Table 6.** *In vitro* screening for genes associated with antibiosis genes

	Antibiosis gene					
**Bacterial strain**	phlD(2,4-DAPG)	phzCD (phenazine-1-carboxylic acid)	prnD (pyrrolnitrin)	pltC (pyoluteorin)	pltB (pyoluteorin)	hcnBC (hydrogen cyanide)
**TW17**	+	−	+	+	+	+

+, Present; −, absent.

### Production of lytic enzyme activity

#### Chitinase activity

Exochitinase activity in GlcNAc monomers was determined in the three treatments, with F.S (18.0–32.3 U ml^−1^) recorded highest compared to treatments TW17+F .S (7.0–9.3 U ml^−1^) and TW17 (1.3–3.7 U ml^−1^) (Table 7). For GlcNAc-2, the F.S. treatment recorded the lowest chitinase activity compared to the TW17+F .S and TW17 treatments. Furthermore, the highest chitinase activity (GlcNAc-2) was recorded after 72 h (3,160.0 U ml^−1^). An increase in chitinase activity was recorded in TW17+F .S treatment over time (Table 7). Endochitinase activity (GlcNAc-3) was recorded as the highest in treatments TW17 and TW17+F .S. However, there was a decrease in chitinase activity in TW17 treatment and an increase in TW17+F .S treatment from 24 to 72 h.

**Table 7.** Chitinase activity between TW17 and *F. solani*

	Chitinase activity (U ml^−1^)								
	Exochitinase						Endochitinase		
	GlcNAc			GlcNAc-2			GlcNAc-3		
**Time (H**)	TW17	S	TW17+F .S	TW17	S	TW17+F .S	TW17	S	TW17+F .S
24	3.67±0.33^f^	32.33±0.88^a^	7.00±0.58^e^	2544.00±202.21^b^	4.67±0.88^c^	2563.00±55.64^b^	1214.33± 41.95^a^	22.67±1.20^f^	850.67±12.72^d^
48	1.67±0.33^g^	26.33±1.20^b^	7.67±0.67^de^	3084.00± 10.21^a^	6.00± 1.16^c^	2984.00±51.23^a^	959.00± 35.37^cd^	24.00±0.58^f^	1038.33±31.93^bc^
72	1.33±0.33^g^	18.00±0.58^c^	9.33±0.33^d^	2625.67±68.37^b^	3.00± 0.58^c^	3160.00±35.12^a^	709.00±95.25^e^	21.00±0.58^f^	1108.67±6.12^ab^
C.V		9.4			7.02			10.08	
LSD		1.93			227.2			114.24	
P>		0.0001			0.0001			0.0001	

The values indicate that the mean of three duplicates in duplicate (*n*=6) with the same superscript letter(s) within the same column is not significantly different by Fisher’s test. According to Fisher’s LSD test (*P*<0.05), different superscript letters (a, b, c and d) indicate significantly different means within a column. *N*-Acetyl-*β*-d-glucosaminide (GlcNAc), and N,N′-diacetyl-*β*-d-chitobioside (GlcNAc-2) for exochitinase activity and *β*-d-N,N′,N″-triacetylchitotriose (GlcNAc-3) for endochitinase activity. Data are presented as mean±standard error.

#### Chitosanase activity

The chitosanase activity detected in TW17+F .S treatment was higher compared to TW17 regardless of time. In all treatments, chitosanase activity increased from 24 to 72 h, with the highest inhibition zone (29.0±1.15) recorded in treatment TW17+F .S. at 72 h (Table 8).

**Table 8.** Chitosanase activity between *F. solani* and TW17

	Chitosanase activity (mm)	
**Time (H**)	TW17	TW17+*F. solani*
24	12.67±0.33^d^	15.00±0.58^cd^
48	14.00±0.58^d^	21.33±0.88^b^
72	17.33±0.88^c^	29.00±1.15^a^
C.V	7.43	
LSD	2.41	
*P*>	0.0001	

The values indicate that the mean of three duplicates in duplicate (*n*=6) with the same superscript letter(s) within the same column is not significantly different by Fisher’s test. According to Fisher’s LSD test (*P* < 0.05), different superscript letters (a, b, c and d) indicate significantly different means within a column. Data are presented as mean ± standard error.

### *In vivo* antagonistic potential of selected TW17 using pot assays

**Fig. 5.**
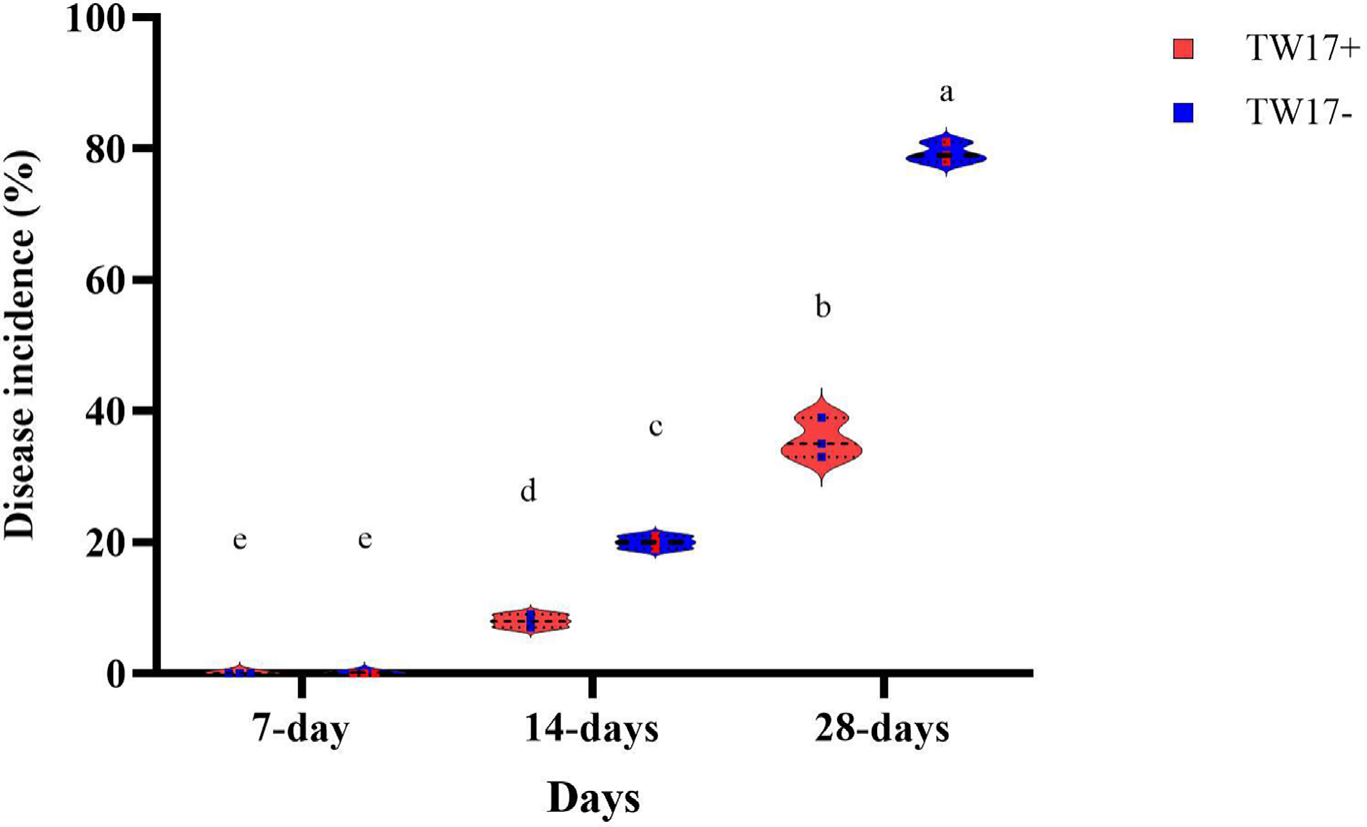
Percentage of disease incidence based on symptomatic plants per total number of plants observed infected with *F. solani* on the root of common beans, TW17+, TW17 treated; TW17-, not treated. According to Fisher’s LSD test (*P* < 0.05), different superscript letters (a, b, c and d) indicate significantly different means within each parameter. The error bars indicate standard error (se).

To assess the percentage of disease incidence, plants treated and untreated with TW17 were infected with *F. solani* to determine the protective effect of the bacterial strain of TW17 (Fig. 5). In both treatments, disease symptoms were detected after 7 days of fungal infection, with untreated plants recording the highest disease incidence compared to treated plants. After 14 days, the untreated plant had a sudden increase of 79.3% in the incidence of the disease compared to the treated plant, which recorded 35.6% (Fig. 5).

**Fig. 6.**
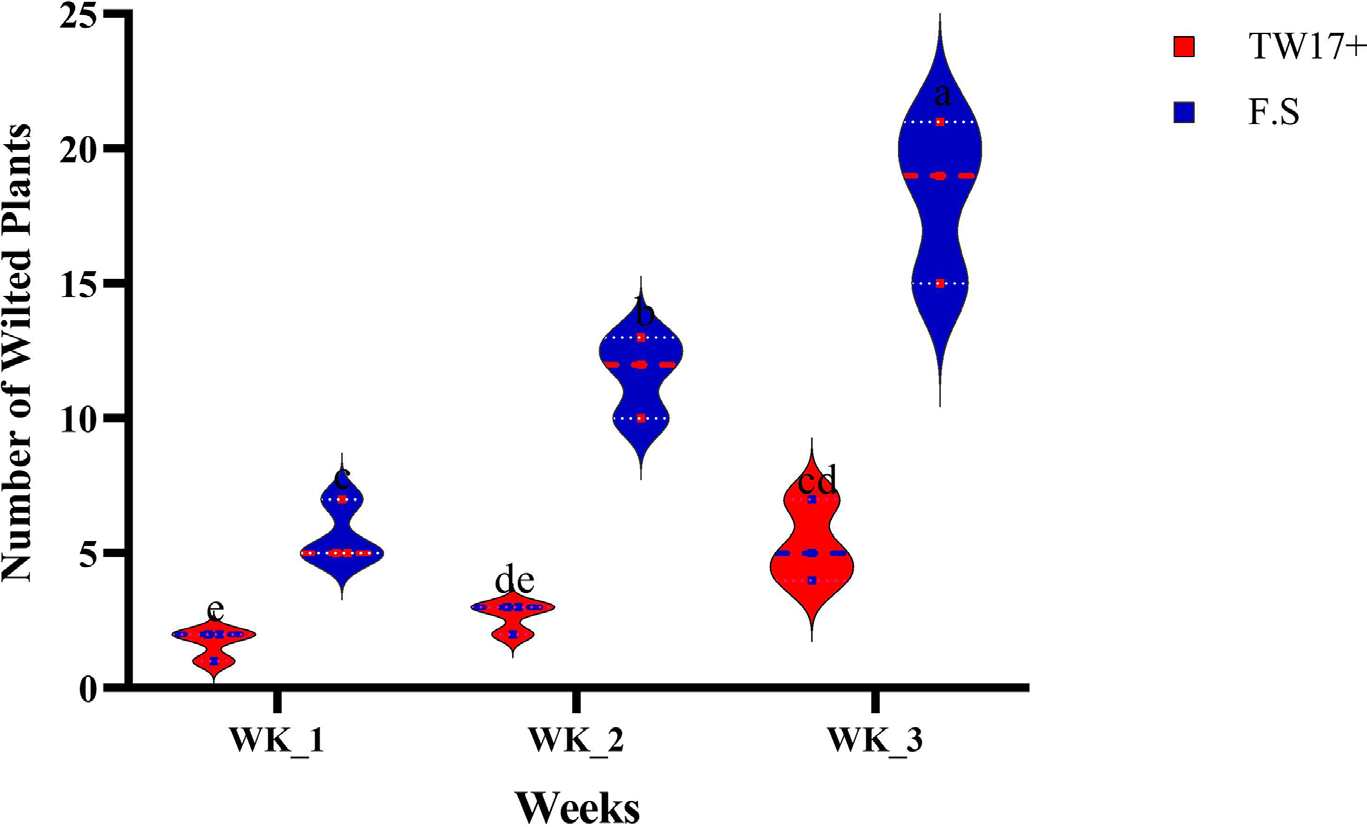
Number of wilted plants observed for 3 weeks, TW17+, TW17 bacterial strain treated; F.S-, *F. solani* treated. According to Fisher’s HSD test (P < 0.05), different superscript letters (a, b, c and d) indicate significantly different means within each parameter. The error bars indicate standard error (se).

Fig. 6 shows data on the number of wilted plants from two treatments, showing the effect of TW17 strain inoculation on the root of common beans infected with *F. solani* after 21 days. The untreated plants (only F.S) showed the highest number of wilted plants from week 1 to week 3 compared to the treated plants (TW17+). The treated plants, on the other hand, exhibited high protection against *F. solani*, with a low number of wilted plants recorded throughout the period (Fig. 6). The number of wilted plants recorded in the period increased significantly, with week 3 recording the highest number (18.0). For the treated plants, there were no significant differences between week 1 and week 2, and there were no significant differences between week 2 and week 3. However, there was a significant difference between week 1 and week 3, with week 3 recording slightly more wilted plants (5.0) than week 2 (3.0) and week 1 (2.0).

### Survival and population density of the TW17 strain in common bean roots

**Fig. 7.**
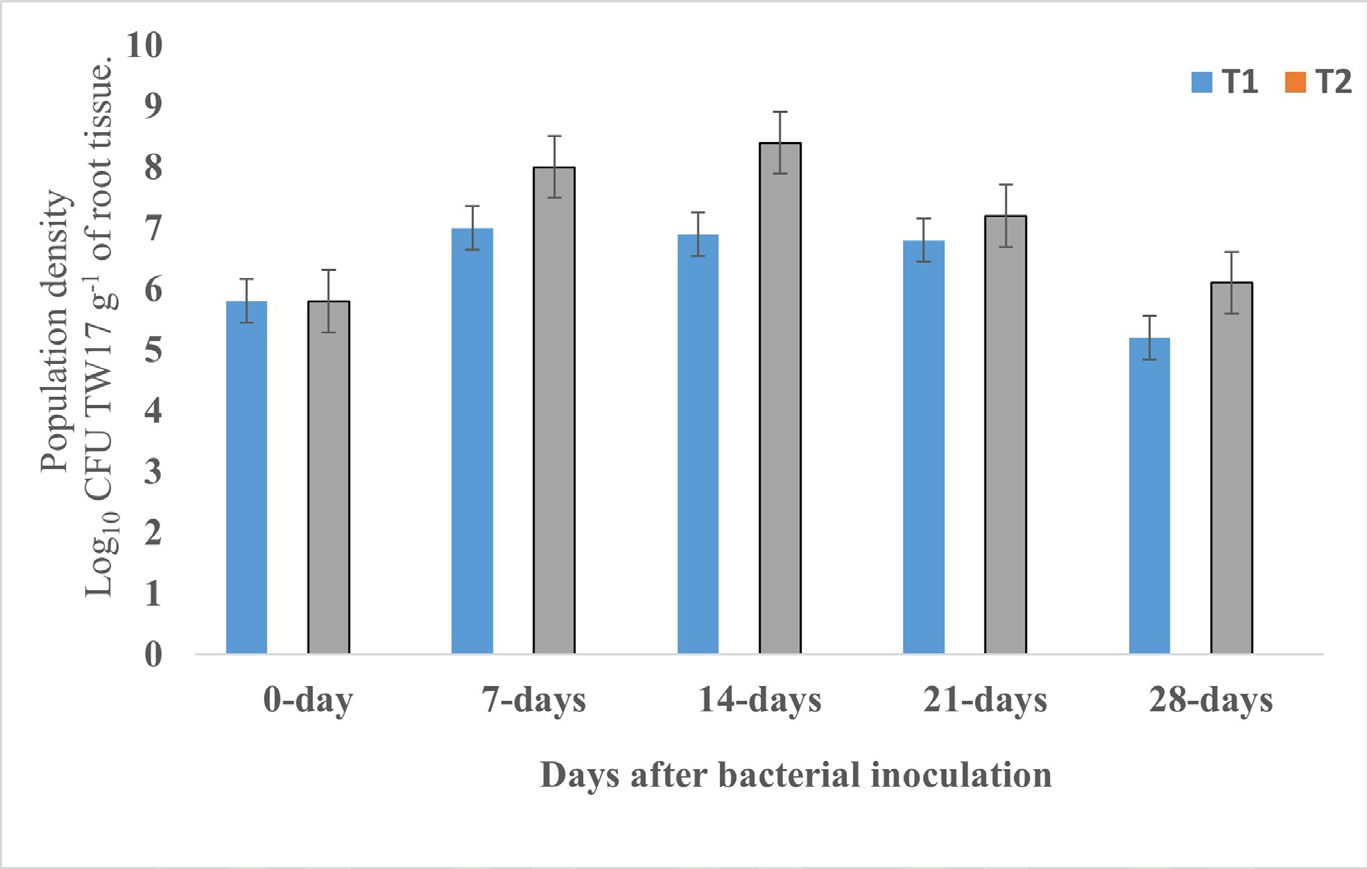
Population density of the TW17 strain on common bean root in greenhouse determined by the c.f.u. counting method. Line T1, c.f.u. of fluorescent TW17 strain in treatment with TW17+*F. solani* treatment; T2, treatment with *F. solani* only treatment; T3, c.f.u. of TW17 strain in treatment with TW17 only treatment; T4, absolute control (without pathogen or bacteria strain). The error bars indicate the standard.

The population density of the TW17 strain on the roots of common beans was determined by the c.f.u. counting method Fig. 7. Lines T_1_ and T_3_ showed the overall c.f.u. counts under fluorescents at the roots of treatments TW17+*F. solani* and TW17 only, respectively. c.f.u. counts (Log_10_) ranged from 5.8 to 7.0 for T_1_ treatment and 5.8 to 8.4 for T_3_ treatment T_3_. However, treatments with T_2_ and T_4_ recorded 0 c.f.u. However, the number of c.f.u. increased for the first 14 days after inoculation and then started to decline for the T_1_ and T_3_ treatments (Fig. 7).

## Discussion

The use of soda lake microbes as a potential biocontrol against phytopathogens has been reported by Wekesa et al. [39]. Despite that, soda lake-derived bacteria offer promising biocontrol potential due to their resilience in extreme environments, making them effective in harsh soils while reducing reliance on chemical pesticides [42,43]. However, challenges such as potential ecological disruption, limited adaptability to non-alkaline soils and regulatory hurdles related to biosafety and ethical bioprospecting have hindered soda lake exploitation as an alternative biocontrol agent [44]. This study explored the potential of *Pseudomonas fluorescens* from Lake Bogoria as a potential biocontrol agent in the management of *F. solani* in common beans. In our study, 10 of the 30 isolates had a varied mycelium inhibition rate of the phytopathogen tested. Among them, isolates TW17 and TW19 had the highest antagonistic activity of >40%. Our results reiterate findings by Dugassa *et al.* [45], who reported the cell-free culture filtrate of *Pseudomonas fluorescens* AAUPF62 inhibiting mycelium growth of *Fusarium* by 71.11%.

Additionally, studies on mycelium inhibition due to the synthesis of lytic enzymes produced by *Pseudomonas* spp. bacteria involved in cell degradation during antagonism were reported by Nehra *et al.* [29]. Mycelium inhibition rate may be due to diffusible bacterial inhibitory antibiosis substances plausibly causing suppression and restriction of the pathogen growth [46]. The results of confrontation were found to be inducible to degrade the cell wall between 48 and 72 h of their direct interaction. The results corroborate previous findings by Trejo-Raya *et al.* [47] on the ability of microbes to degrade cell walls in direct contact with the pathogen.

The results based on 16S rRNA; blast confirms the taxonomical identity of TW19 as *Pseudomonas fluorescens*. However, the isolate TW17 aligned with *Pseudomonas* with a 97.5% identity, making it a unique isolate. These results are similar to previous reporting of *Pseudomonas* sp. (*Gammaproteobacteria*) from soda lakes [48] who reported *Gammaproteobacteria* as dominant class in soda lakes.

From the study, the strain TW17 was further characterized to determine the antibiosis genes present. Our results showed the presence of genes involved in the biosynthesis of secondary metabolites such as *hcn*, *plt*, *prn* and *phl*, which are involved in the production of hydrogen cyanide, pyoluteorin, pyrrolnitrin and 2,4-DAPG, respectively [49]. According to Pellicciaro *et al.* [50], pyoluteorin is produced by *Pseudomonas* spp. Furthermore, our results agree with the previous findings by Balthazar *et al.* [51], who reported that *Pseudomonas* spp. harbour *plt* gene clusters responsible for the biosynthesis of secondary metabolites.

The production of lytic enzymes such as chitinase and chitosanase was evaluated as they play a big role in the fungal lysate [52]. Our results showed the production of exochitinase and endochitinase activities at 24 h, 48 h and 72 h in treatment with TW17+F .S, which resulted in cell degradation. The function of endochitinase was to cleave the chitin chain at the internal site in the fungal cell wall to generate the substrate for subsequent exochitinase activity. According to Barroso-Solares *et al.* [53], chitin and chitosan are the major components of the cell wall in some filamentous fungi. Chitosan, which is highly deacetylated from chitin, helps the fungus to protect itself [54]. Bacterial production of chitosanase facilitates the degradation of chitosan, and in conjunction with chitinase, it acts synergistically to break down the fungal cell wall [53]. Therefore, it is possible that TW17 lytic enzymes could have caused a decrease in the number of hyphae when bacteria confronted the fungus. The cell wall was visualized under a light microscope from the confrontation assay experiment, where less branching of hyphae and abnormal growth of the *F. solani* could be seen. Similar results have also been reported by Báez-Astorga *et al.* [37] on the test of *Bacillus* spp. against *R. solani*.

During the *in vivo* assay, the persistence and concentration of TW17 on the roots of common beans were determined using the culture method. Our results demonstrated the presence of the majority of fluorescent *pseudomonas* sp. from the treated roots compared to the untreated plants. Therefore, TW17 was capable of surviving in the root system of common beans even after infection with phytopathogens. The results were consistent with the previous findings reported by Bakki *et al.* [55]. The colony count obtained was also consistent and reached the threshold by which *Pseudomonas* spp. was required for its effectiveness in disease suppression.

Our results on disease incidence showed the ability of TW17 to exhibit high protection against *F. solani*. Furthermore, the number of wilted plants was also recorded at its lowest compared to treatment with only *F. solani*. Similar findings have been reported by Bakki *et al.* [55], in which microbe inoculation is able to protect the root against plant pathogens. The ability to induce a host defence system by microbes is also attributed to microbes to defend against diseases [56].

In conclusion, *Pseudomonas* spp. from Lake Bogoria had significant antagonistic activity against *F. solani* in dual culture and confrontation techniques. Additionally, they possess antibiotic-associated genes and lytic enzymes (chitinase and chitosanase) responsible for suppressing mycelium growth. *In vivo* assay, the TW17 strain showed significant disease control and lower incidence of wilt. Additionally, the colony counts in persistence and survival showed the ability to colonize the root system of the plant despite the presence of the pathogen. The use of soda lake-derived bacteria in biocontrol faces limitations such as strain specificity, scalability challenges in mass production and reduced effectiveness in non-extreme environments. Future research should focus on genome-guided strain optimization, formulation development for broader adaptability and field trials to evaluate long-term ecological impact and efficacy.

## Abbreviations

blast: basic local alignment search tool
CDA: chitosanase detection agar
2,4-DAPG: 2,4-diacetyl phloroglucinol
LB: Luria–Bertani
LSD: least significant difference
NCBI: National Center for Biotechnology Information
SDA: Sabouraud dextrose agar

## References

[1] Babirye I, Nakazi F, Birachi EA, Wabbi JB, Ugen MA (2023). Exploring processed common beans market in Kenya: Implications for the business community.

[2] Castro-Guerrero NA, Isidra-Arellano MC, Mendoza-Cozatl DG, Valdés-López O (2016). Common bean: a legume model on the rise for unraveling responses and adaptations to iron, zinc, and phosphate deficiencies. Front Plant Sci.

[3] Rathna Priya TS, Manickavasagan A (2020). In Pulses: Processing and Product Development.

[4] Celmeli T, Sari H, Canci H, Sari D, Adak A (2018). The nutritional content of common bean (*Phaseolus vulgaris* L.) landraces in comparison to modern varieties. *Agronomy*.

[5] Uebersax MA, Cichy KA, Gomez FE, Porch TG, Heitholt J (2023). Dry beans (phaseolus vulgaris l.) as a vital component of sustainable agriculture and food security—a review. Legum Sci.

[6] Muteti K, Wambua S, Gichangi A, Mutua M (2022). The household income determinants crop sales: the case of common bean production and marketing in selected bean corridors in Kenya. African J Rural Dev.

[7] Moorthy PNK, Kumar NRP, Mani M, Saroja S (2022). Pests and their management in leguminous vegetables. Trends Hortic Entomol.

[8] Mani M (2022). Pest management in horticultural crops under protected cultivation. Trends Hortic Entomol.

[9] Duku C, Groot A, Demissie T, Muhwanga J, Nzoka O (2020). Common beans Kenya: Climate risk assessment.

[10] Zitnick-Anderson K, Oladzadabbasabadi A, Jain S, Modderman C, Osorno JM (2020). Sources of resistance to *Fusarium solani* and associated genomic regions in common bean diversity panels. Front Genet.

[11] Gherbawy YA, Hussein MA, Hassany NA, Shebany YM, Hassan S (2021). Phylogeny and pathogenicity of *Fusarium solani* species complex (FSSC) associated with potato tubers. J Basic Microbiol.

[12] Chenari S, Abbasi S, Chehri K (2024). Phylogeny and host specificity of *Fusarium solani* species complex isolated from chickpea, lentil and common bean. Arch Phytopathol plant Prot.

[13] Pierre E, Fabiola YN, Vanessa ND, Tobias EB, Marie-claire T (2023). The co-occurrence of drought and *Fusarium solani* f. sp. Phaseoli Fs4 infection exacerbates the *Fusarium* root rot symptoms in common bean (*Phaseolus vulgaris* L.). Physiol Mol Plant Pathol.

[14] Araka A, Muraya MM, Kuria EK (2025). Prevalence of bean root rot pathogens in tharaka Nithi County, Kenya. J Sci Technol.

[15] Moparthi S, Burrows M, Mgbechi-Ezeri J, Agindotan B (2021). *Fusarium* spp. associated with root rot of pulse crops and their cross-pathogenicity to cereal crops in Montana. *Plant Dis*.

[16] Ashraf H, Anjum T, Riaz S, Naseem S (2020). Microwave-assisted green synthesis and characterization of silver nanoparticles using *Melia azedarach* for the management of *Fusarium* wilt in tomato. Front Microbiol.

[17] Larkin RP, Brewer MT (2020). Effects of crop rotation and biocontrol amendments on rhizoctonia disease of potato and soil microbial communities. Agric.

[18] Rani AT, Vasudev K, Pandey KK, Singh B (2020). Sucking pests of vegetable crops. Sucking Pests Crop.

[19] Quesada-Moraga E, González-Mas N, Yousef-Yousef M, Garrido-Jurado I, Fernández-Bravo M (2023). Key role of environmental competence in successful use of entomopathogenic fungi in microbial pest control. J Pest Sci.

[20] Kopp EB, Niklaus PA, Wuest SE (2023). Ecological principles to guide the development of crop variety mixtures. J Plant Ecol.

[21] Monteiro A, Santos S (2022). Sustainable approach to weed management: the role of precision weed management. *Agronomy*.

[22] Wekesa TB, Wekesa VW, Onguso JM, Wafula EN, Kavesu N (2022). Isolation and characterization of *Bacillus* velezensis from Lake Bogoria as a potential biocontrol of *Fusarium solani* in *Phaseolus vulgaris* L. *Bacteria*.

[23] Abeysinghe S (2012). Biological control of *Fusarium solan*i f. sp. phaseoli the causal agent of root rot of bean using *Bacillus subtilis* CA32 and *Trichoderma harzianum* RU01. Ruhuna J Sci.

[24] Lv N, Tao C, Ou Y, Wang J, Deng X (2023). Root-associated antagonistic *Pseudomonas spp*. contribute to soil suppressiveness against banana fusarium wilt disease of banana. Microbiol Spectr.

[25] Gade RM, Koche MD (2022). Enhancing the growth and disease suppression ability of *Pseudomonas fluorescens*. New Futur Dev Microb Biotechnol Bioeng Sustain Agric Adv Microbe-based Biostimulants.

[26] Sun Z, Hu Y, Yang Y-X, Lei M-Y, Han Z-M (2025). Changes in the diversity of ginseng endophyte flora driven by *Fusarium solani*. Front Microbiol.

[27] Jaffar NS, Jawan R, Chong KP (2022). The potential of lactic acid bacteria in mediating the control of plant diseases and plant growth stimulation in crop production - a mini review. Front Plant Sci.

[28] Khalil MSM, Hassan MHAR, Mahmoud AF, Morsy KMM (2022). Involvement of secondary metabolites and extracellular lytic enzymes produced by plant growth promoting rhizobacteria in inhibiting the soilborne pathogens in Faba Bean Plants. *J Trop Plant Pests Dis*.

[29] Nehra S, Gothwal RK, Dhingra S, Varshney AK, Solanki PS (2022). Mechanism of antagonism: hyperparasitism and antibiosis. Microb Biocontrol Sustain Agric Phytopathogen Manag.

[30] Mutungi PM, Wekesa VW, Onguso J, Kanga E, Baleba SBS (2021). Culturable bacterial endophytes associated with shrubs growing along the draw-down zone of lake Bogoria, Kenya: assessment of antifungal potential against *Fusarium solani* and induction of bean root rot protection. Front Plant Sci.

[31] Tamiru G, Muleta D (2018). The effect of rhizobia isolates against black root rot disease of faba bean (Vicia faba L) caused by *Fusarium solani*. Open Agric J.

[32] Atlas RM (2010). Handb. Microbiol. Media.

[33] Aydi R, Abdallah B, Jabnoun-Khiareddine H, Nefzi A, Mokni-Tlili S (2016). Endophytic bacteria from datura stramonium for fusarium wilt suppression and tomato growth promotion. J Microb Biochem Technol.

[34] Tamura K, Stecher G, Kumar S (2021). MEGA11: molecular evolutionary genetics analysis version 11. Mol Biol Evol.

[35] Falert S, Akarapisan A (2019). Identification of *Fusarium spp*. causing dry rot of seed potato tubers in northern, Thailand. Int J Agric Technol.

[36] Chakravarthi B, Singh S, Kamalraj S, Gupta VK, Jayabaskaran C (2020). Evaluation of spore inoculum and confirmation of pathway genetic blueprint of T13αh and DBAT from a taxol-producing endophytic fungus. Sci Reports.

[37] Báez-Astorga PA, Cázares-Álvarez JE, Cruz-Mendívil A, Quiroz-Figueroa FR, Sánchez-Valle VI (2022). Molecular and biochemical characterisation of antagonistic mechanisms of the biocontrol agent *Bacillus cereus B* 25 inhibiting the growth of the phytopathogen *Fusarium verticillioides* P03 during their direct interaction *in vitro*. Biocontrol Sci Technol.

[38] Fawzya YN, Chasanah E (2022). Isolation of chitinolytic enzymes and development of chitooligosaccharides in Indonesia. Chitooligosaccharides Prev Control Dis.

[39] Wekesa TB, Wafula EN, Kavesu N, Sangura RM (2023). Taxonomical, functional, and cytopathological characterization of *Bacillus spp*. from Lake Magadi, Kenya, against *Rhizoctonia* solani Kühn in *Phaseolus vulgaris* L. J Basic Microbiol.

[40] Bock CH, Chiang KS, Del Ponte EM (2022). Plant disease severity estimated visually: a century of research, best practices, and opportunities for improving methods and practices to maximize accuracy. Trop Plant Pathol.

[41] Moruzzi S, Firrao G, Polano C, Borselli S, Loschi A (2017). Genomic-assisted characterisation of *Pseudomonas* sp. strain Pf4, a potential biocontrol agent in hydroponics. Biocontrol Sci Technol.

[42] Kiama CW, Njire MM, Kambura AK, Matiru VN, Wafula EN (2020). Isolation and characterization of bacteria from lakes olbolosat and oloiden, kenya. African J Microbiol Res.

[43] Nyakeri EM, Mwirichia R, Boga H (2018). Isolation and characterization of enzyme producing bacteria from Lake Magadi, an extreme soda lake in Kenya. JMEN.

[44] Kochhar N, I K K, Shrivastava S, Ghosh A, Rawat VS (2022). Perspectives on the microorganism of extreme environments and their applications. *Curr Res Microb Sci*.

[45] Dugassa A, Alemu T, Woldehawariat Y (2021). In-vitro compatibility assay of indigenous *Trichoderma* and *Pseudomonas species* and their antagonistic activities against black root rot disease (Fusarium solani) of faba bean (Vicia faba L.). BMC Microbiol.

[46] Toghueo RMK, Eke P, Zabalgogeazcoa Í, de Aldana BRV, Nana LW (2016). Biocontrol and growth enhancement potential of two endophytic *Trichoderma spp*. from *Terminalia* catappa against the causative agent of common bean root rot (*Fusarium solani*). Biological Control.

[47] Trejo-Raya AB, Rodríguez-Romero VM, Bautista-Baños S, Quiroz-Figueroa FR, Villanueva-Arce R (2021). Effective in vitro control of two phytopathogens of agricultural interest using cell-free extracts of *Pseudomonas fluorescens* and Chitosan. *Molecules*.

[48] Pellegrinetti TA, Cotta SR, Feitosa YB, Melo PLA, Bieluczyk W (2024). The role of microbial communities in biogeochemical cycles and greenhouse gas emissions within tropical soda lakes. Sci Total Environ.

[49] Mikiciński A, Puławska J, Molzhigitova A, Sobiczewski P (2022). Evaluation of antagonistic mechanisms of bacterial species recognized for the first time for their biocontrol activity against fire blight (erwinia amylovora).

[50] Pellicciaro M, Padoan E, Lione G, Celi L, Gonthier P (2022). Pyoluteorin produced by the biocontrol agent *Pseudomonas protegens* is involved in the inhibition of *Heterobasidion* species present in Europe. *Pathogens*.

[51] Balthazar C, St-Onge R, Léger G, Lamarre SG, Joly DL (2022). Pyoluteorin and 2,4-diacetylphloroglucinol are major contributors to *Pseudomonas protegens* Pf-5 biocontrol against *Botrytis cinerea* in cannabis. Front Microbiol.

[52] Zhao P, Li P, Wu S, Zhou M, Zhi R (2019). Volatile organic compounds (VOCs) from *Bacillus subtilis* CF-3 reduce anthracnose and elicit active defense responses in harvested litchi fruits. AMB Express.

[53] Barroso-Solares S, Lopez-Moya F, Fraile T, Prieto ÁC, Lopez-Llorca L (2025). Chitin and chitosan quantification in fungal cell wall via Raman spectroscopy. Spectrochim Acta A Mol Biomol Spectrosc.

[54] Tamrela H, Sugiyanto A, Santoso I, Fadhilah QG (2021). The qualitative screening of cellulolytic, chitinolytic, IAA-producing, and phosphate solubilizing bacteria from black soldier fly larvae (*Hermetia illucens L*.). IOP Conf Ser: Earth Environ Sci.

[55] Bakki M, Banane B, Marhane O, Esmaeel Q, Hatimi A (2024). Phosphate solubilizing *Pseudomonas* and *Bacillus* combined with rock phosphates promoting tomato growth and reducing bacterial canker disease. Front Microbiol.

[56] Wilson SK, Pretorius T, Naidoo S (2023). Mechanisms of systemic resistance to pathogen infection in plants and their potential application in forestry. BMC Plant Biol.

[57] Raaijmakers JM, Weller DM, Thomashow LS (1997). Frequency of antibiotic-producing *Pseudomonas* spp. in natural environments. Appl Environ Microbiol.

[58] Souza JT, Raaijmakers JM Polymorphisms within the *prnD* and *pltC* genes from pyrrolnitrin and pyoluteorin-producing *Pseudomonas* and *Burkholderia* spp. FEMS Microbiol Ecol.

[59] Mavrodi OV, McSpadden Gardener BB, Mavrodi DV, Bonsall RF, Weller DM (2001). Genetic diversity of phlD from 2,4-diacetylphloroglucinol-producing fluorescent *Pseudomonas* spp. Phytopathology.

[60] Ramette A, Frapolli M, Défago G, Moënne-Loccoz Y (2007). Phylogeny of HCN synthase-encoding hcnBC genes in biocontrol fluorescent *Pseudomonads* and its relationship with host plant species and HCN synthesis ability.

